# UNC-42 function is required for the ectopic expression of ASH markers in *mls-2* mutant animals

**DOI:** 10.17912/micropub.biology.000116

**Published:** 2019-06-12

**Authors:** Jordan F. Wood, Denise M. Ferkey

**Affiliations:** 1 Department of Biological Sciences, University at Buffalo, The State University of New York, Buffalo, NY USA 14260

**Figure 1. Ectopic ASH gene expression in mls-2(cc615) mutant animals requires unc-42:**
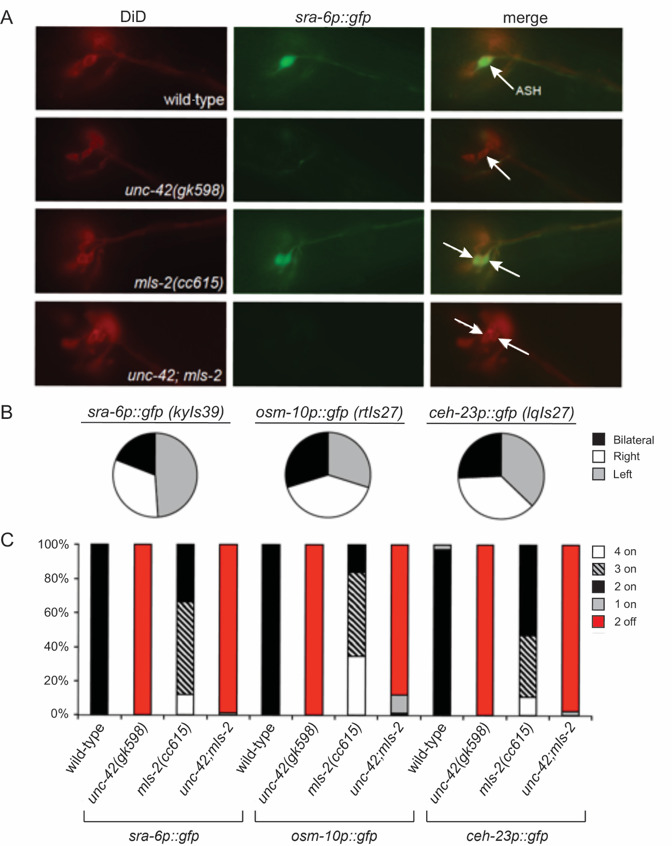
UNC-42 function is required for the ectopic expression of ASH terminal differentiation gene markers in the ectopic ASH-like cells of *mls-2* loss-of-function animals. The native ASHs and ectopic ASH-like neurons were identified by positional labeling with the lipophilic dye DiD, as previously described (Perkins*et al.,* 1986). (A) Representative images of *sra-6p::gfp* are shown. Native ASH neurons of wild-type and *unc-42* animals are indicated by white arrows. Native and ectopic ASH-like cells are indicated for *mls-2* and *unc-42;mls-2* animals. (B) Pie charts indicate the degree of bilateral ectopic marker expression in *mls-2(cc615)* animals, as well as right versus left marker expression in the cases of unilateral ectopic expression. (C) The percentage of animals with marker expression in 4, 3, 2, 1 and zero (2 off) ASH and ASH-like cells, combined, is shown. n > 34 animals were examined for each genotype.

## Description

The HMX/NKS homeodomain transcription factor MLS-2 is required to initiate the expression of the AWC terminal selector *ceh-36*, and as such the AWCs of *mls-2* loss-of-function mutant animals fail to express downstream AWC-specific terminal differentiation genes (Kim *et al.,* 2010). Interestingly, loss of *mls-2* function was also shown to result in ectopic expression of ASH markers (*sra-6p::gfp* and *osm-10p::gfp*) in at least one neuron in a large percentage of *mls-2* mutant animals (Kim *et al.,* 2010). The cells that ectopically express the ASH markers are adjacent to the native ASHs and, like native ASH neurons, dye-fill with lipophilic dyes such as DiD (Perkins *et al.,* 1986; Kim *et al.,* 2010).

Since ASH expression of both *sra-6p::gfp* (Baran *et al.,* 1999; Wood and Ferkey, 2019) and *osm-10p::gfp* (Wood and Ferkey, 2019), as well as *ceh-23p::gfp* (Wood and Ferkey, 2019), depends upon the paired-like homeodomain transcription factor UNC-42, we assessed whether the ectopic expression of these ASH markers requires UNC-42 function as well. We first examined the expression pattern of stably integrated reporters for *sra-6p::gfp*, *osm-10p::gfp* and *ceh-23p::gfp* in *mls-2(cc615)* loss-of-function mutant animals. In addition to being expressed in the native ASH neurons, for each transgene we confirmed ectopic marker expression unilaterally or bilaterally in the ectopic ASH-like cells of *mls-2(cc615)* mutant animals, which were identified by dye-filling (Figure 1). We note that there was no obvious directional bias as to which side of the bilateral ASH-like pair the unilateral ectopic expression arose (Figure 1B). We found that both native and ectopic expression of all three ASH markers was lost in the *unc-42(gk598)*;*mls-2(cc615)* double mutants, although the native and ectopic cells retained dye-filling capacity (Figure 1A, C). Thus, the ectopic expression of these ASH markers in the absence of MLS-2 function depends upon UNC-42, as native ASH expression does (Baran *et al.,* 1999; Wood and Ferkey, 2019).

## Reagents

DiD was purchased from Molecular Probes (Invitrogen).

The VC1444 *unc-42(gk598)* strains was generated by the *C. elegans* Reverse Genetics Core Facility at the University of British Columbia, which is part of the International *C. elegans* Gene Knockout Consortium. The *gk598* allele contains a 1430 basepair deletion (898 basepairs of 5’ UTR sequence, exon 1 and 481 basepairs of intron 1). VC1444 was outcrossed 6x to N2 to generate FG498 (Wood and Ferkey, 2019).

Strains used in this study include: N2 Bristol wild-type, FG498 *unc-42(gk598)*, LW227 *mls-2(cc615)*, FG746 *unc-42(gk598);mls-2(cc615)*, CX3465 *kyIs39 [sra-6::gfp + lin-15(+)]*, FG750 *unc-42(gk598);kyIs39*, FG749 *mls-2(cc615);kyIs39*, FG748 *unc-42(gk598);mls-2(cc615);kyIs39*, HA1695 *rtIs27 [osm-10p::gfp]*, FG573 *unc-42(gk598);rtIs27*,FG745 *mls-2(cc615);rtIs27*,FG747*unc-42(gk598);mls-2(cc615);rtIs27*, LE732 *lqIs27 [ceh-23::gfp + lin-15(+)]*, FG839 *unc-42(gk598);lqIs27*, FG840 *mls-2(cc615);lqIs27*,FG841 *unc-42(gk598);mls-2(cc615);lqIs27*. Some of the strains used in this study were obtained from the *Caenorhabditis* Genetics Center, which is funded in part by the National Institutes of Health – National Center for Research Resources. Strains generated in our lab for this study have not been sent to the CGC, but are available by request.
